# Hospital admissions among adolescents with local authority care experience or special educational needs in England: a population-based cohort study using linked administrative data from health, education and social care services

**DOI:** 10.1136/bmjph-2025-003106

**Published:** 2026-03-12

**Authors:** Ruth M Blackburn, Theodora Kokosi, Michelle Heys, Ruth Gilbert, Ania Zylbersztejn

**Affiliations:** 1 UCL Great Ormond Street Institute of Child Health, University College London, London, UK; 2 Population Health Improvement UK (PHI-UK), London, UK; 3 University College London Institute for Global Health, London, UK; 4 NIHR Great Ormond Street Hospital Biomedical Research Centre, London, UK

**Keywords:** Adolescent, Public Health, Epidemiology

## Abstract

**Introduction:**

Children and young people in state care or with special educational needs (SEN) experience disproportionately higher rates of long-term physical and mental illness compared with their peers. However, few large-scale studies have explored the intersection of social care, education systems and healthcare services during adolescence. We aimed to quantify planned and unplanned hospital admissions during adolescence for pupils with SEN and/or experience of state care in England.

**Methods:**

We analysed linked administrative records from hospital, education and social care systems for adolescents in England using the Education and Child Health Insights from Linked Data (ECHILD) database. The cohort comprised pupils starting secondary school between 2007–2008 and 2011–2012 (aged 11 years), with follow-up until March 2020 (aged 18–23 years). Hospital admission rates were examined by gender and age across six mutually exclusive groups reflecting statutory support during school: (1) no support, (2) school-based SEN support only, (3) formalised SEN support only (education, health and care plan; EHCP), (4) care-experienced only, (5) SEN support and care-experienced and (6) EHCP and care-experienced. Rate ratios were estimated for each group relative to no support using negative binomial regression models.

**Results:**

The cohort included 2 807 230 pupils: 64% (1 791 190) received no support, 31% (876 400) received SEN support only, 3% (84 410) received EHCP only and 1% (29 330) received SEN support and care experience, with the remaining groups accounting for <1%. Unplanned admissions were higher in all groups receiving support than peers with no support and were highest in care-experienced girls with an EHCP (21.9 (95% CI 21.4 to 22.4)/100 person-years (100PY)). Mental health-related admissions accounted for 48% of all unplanned admissions in girls and 33% in boys. Pregnancy-related admission rates were highest in care-experienced girls (14/100PY).

**Conclusions:**

We found evidence of high levels of unplanned admissions coupled with low levels of planned care for pupils with multiple needs, indicating a need for preventative care.

**WHAT IS ALREADY KNOWN ON THIS TOPIC**

- Previous research has established that children and young people who are care-experienced—such as those in foster care or with involvement in the child welfare system—and/or who have additional special educational needs (SEN) experience a disproportionately high burden of physical and mental health challenges. Although these young people are known to multiple service systems, concerns persist about the accessibility, co-ordination and overall effectiveness of their care, which is often fragmented across sectors and disrupted during transitions from child and adult services.

**WHAT THIS STUDY ADDS**

- This research takes a multi-sectoral approach to quantify both planned and unplanned hospital admissions among adolescents in England who are care-experienced and/or have additional SEN. The findings identify high rates of unplanned hospital use and low levels of planned use among adolescents with intersecting needs, indicating missed opportunities for preventative and co-ordinated interventions.

**HOW THIS STUDY MIGHT AFFECT RESEARCH, PRACTICE OR POLICY**

- The findings highlight the complex and intersecting needs of young people across physical, mental and reproductive health domains, as well as education and social care systems. The study provides evidence to support more integrated, proactive and early intervention strategies and underscores the potential benefits of improved co-ordination across health, education and social care service sectors.

## Introduction

 Disparities in healthcare access, health outcomes and the social determinants of health are a global concern for child development.^1^ Children and young people in state care or with special educational needs (SEN) more frequently have additional long-term physical and mental health needs than their peers.^1^,^3^ However, few large-scale studies in the UK have examined the intersections between health, social care and education service provision required to gain more holistic insight into the complexity and multifaceted needs of these young people.^4^

In England, by the age of 18 years, approximately 3%–4% of children have experienced state care—typically in out-of-home settings such as foster care or with extended family—and are termed children looked after (CLA).^5^ These care-experienced children more frequently experience adverse childhood experiences such as physical, sexual or emotional abuse and have poorer long-term health, education and employment outcomes than peers who never experience out-of-home care.^6^ SEN provision refers to additional support for children with health, learning or behavioural problems that impact their ability to learn. In England, over a third of children receive SEN provision during school age,^7^ including those with an education, health and care plan (EHCP). An EHCP involves a formal assessment requested by the school or parents, followed by a plan of care and additional funding for support up to the age of 25 years. In contrast, SEN support (formerly School Action/Plus) is less formalised, often inconsistent, funded by a block notional budget to schools allocated based on a national formula^8^ and is given at the discretion of the school as part of the usual curriculum (eg, extra help with communication or physical needs, special learning programmes). Social care and educational needs are often linked; by Year 11 (age 16), over 80% of children who have ever experienced care have SEN recorded, and 23% have an EHCP.^7^

Adolescents experience multiple transitions and changes, including ageing out of children’s services for health, education and social care that are structured to provide greater continuity and holistic care than adult services.^9^ Service transitions are typically associated with marked rises in unplanned hospital admissions for physical and mental causes among young people aged 16–18 years.^10^ Health in adolescence is an important indicator of adult health, with three-quarters of lifetime mental health conditions having onset by the age of 25 years.^11^ Poor childhood mental health is a particular concern, with growing recognition that community services for children and young people are unable to meet demand. This affects the use of acute hospital care among young people who are admitted to hospitals. For example, in 2021–2022, almost 1 in 5 paediatric unplanned hospital admissions for 11–15-year-olds were for mental illness or self-harm, with much higher rates among girls than boys.^12^ In children and young people, manifestations of stress/distress indicative of mental health concerns can also present more broadly as physical symptoms (eg, medically unexplained pain), as well as behavioural symptoms, including self-harm or substance misuse.^13^

While it is known that young people receiving support for social care or SEN have greater health needs, there is limited evidence quantifying how the use of healthcare services changes during the adolescent period for these groups. Previous research suggests that children and young people with SEN or who are care-experienced less frequently receive appropriate care in community settings,^1 9 14^ likely contributing to poorer long-term physical and mental health.^6 15 16^ Understanding patterns of health service demand within population groups, and over time, can help inform intervention points and service configuration.^917^,^19^ We, therefore, aimed to quantify planned and unplanned (emergency) hospital admissions among groups of adolescents in England with anticipated differences in health, education and social care needs, as indicated by records of care experience and/or having SEN provision, within strata defined by age and gender. We also investigated the subset of unplanned hospital admissions indicative of mental health, stress/distress or related psychosocial needs, which are important indicators of urgent mental health needs, with rising rates for children and young people in England.^12^ Admissions of this type are extremely distressing for a young person, expensive and inefficient for health services and, in some instances, may be preventable through earlier intervention and support in community settings.^20^

## Methods

### Data source

We used the Education and Child Health Insights from Linked Data (ECHILD) database of de-identified administrative health, education and social care records for all children and young people in England.^21 22^ Health data from Hospital Episode Statistics captures details of all National Health Service-funded hospital admissions,^23^ including patient demographics and International Classification of Diseases (ICD-10) coded diagnostic data. Information about pupil characteristics and enrolment in all state-funded education (93% of all pupils) is captured in termly school censuses from the National Pupil Database,^24^ including linked information on CLA status.^25^ Data availability changes over time, resulting in different follow-up durations for pupils of different ages (online supplemental appendix figure 1).

### Study population

The study cohort included all pupils aged 11 years at the start of the academic years 2007–2008 to 2011–2012 (born between 1996–1997 and 2000–2001) who were enrolled in state-funded schools in England in Year 7 (or in specialist provision and not following the National Curriculum). We excluded pupils with unrecorded gender (n=34). Children and young people were followed up from 1 September of Year 7 to 1 March 2020, their 24th birthday, or death, whichever occurred first. We were unable to identify or make analytical adjustments for pupils who migrated out of the country after school enrolment.

### Exposures

We indicated the highest level of SEN provision (EHCPs, including attending specialist provision, SEN support and none) ever recorded in any school census during primary school (Years 1–6). CLA records for children aged <5 years who have not started school are under-ascertained within ECHILD because the pseudo-identifiers for these records cannot be reliably linked to subsequent education records for the same child. We, therefore, applied a broader time window for identifying care-experienced children, defined as a CLA record(s) at any point before the end of secondary school (31 August of the year they turn 16 years), to increase ascertainment and because experiences of adversity likely predate the first recorded care placement and may have enduring health impacts.

We derived six mutually exclusive groups of children with varying levels of statutory support from education (three levels—none, SEN support and EHCP) and social care (two levels—CLA and not CLA): no statutory support, SEN support only, EHCP only, CLA only, both EHCP and CLA and both SEN support and CLA. We hypothesised that groups with greater complexity and intensity of support (eg, EHCP vs SEN support) would also have additional healthcare demand.

### Characteristics and covariates

We extracted information on baseline (spring term, Year 7) pupil characteristics from the National Pupil Database, including month/year of birth, gender, free school meals eligibility (FSM), English as first language, Income Deprivation Affecting Children Index quintile of area-level deprivation and ethnic group (as defined in table 1). We used ICD-10 codes recorded during hospital admissions from 1 April 1997 to 1 September of Year 7 to identify children with a chronic condition likely to require follow-up in healthcare for more than 1 year.^26^ We describe the proportion of pupils with a chronic condition to capture underlying differences in long-term health between exposure groups. We expected that chronic conditions would be more common among pupils with an EHCP (compared with those without an EHCP) and causally linked to the outcomes under investigation.

**Table 1:** Overview of cohort characteristics

	All children		Any SEN or CLA provision		Only EHCP		Only SEN support		Only CLA		EHCP and CLA		SEN support and CLA	
	N	%	N	%	N	%	N	%	N	%	N	%	N	%
Total (% of all)	2 807 230	100	1 016 040	36	84 410	3	876 400	31	13 260	k	12 660	k	29 330	1
Gender														
Females	1 368 120	49	383 040	38	21 710	26	334 880	38	8940	67	3500	28	14 030	48
Males	1 439 110	51	633 000	62	62 700	74	541 520	62	4320	33	9160	72	15 300	52
Month of birth														
September–October	481 040	17	141 150	14	13 000	15	118 570	14	2710	20	2180	17	4690	16
November–December	459 590	16	148 040	15	13 330	16	125 430	14	2410	18	2150	17	4720	16
January–February	451 920	16	157 260	15	13 510	16	134 490	15	2410	18	2070	16	4780	16
March–April	461 480	16	170 640	17	14 140	17	147 540	17	2070	16	2110	17	4790	16
May–June	469 570	17	188 230	19	14 860	18	164 360	19	1930	15	2080	16	5000	17
July–August	483 620	17	210 720	21	15 570	18	186 010	21	1730	13	2080	16	5340	18
Academic cohort														
2007–2008	565 810	20	202 780	20	16 850	20	175 820	20	2410	18	2460	19	5260	18
2008–2009	576 110	21	203 170	20	17 330	21	174 800	20	2620	20	2760	22	5650	19
2009–2010	562 800	20	201 970	20	16 940	20	173 870	20	2680	20	2540	20	5940	20
2010–2011	557 940	20	204 840	20	16 870	20	176 490	20	2800	21	2530	20	6150	21
2011–2012	544 560	19	203 280	20	16 420	19	175 420	20	2740	21	2370	19	6330	22
Ethnic group														
White	2 258 520	80	807 420	79	68 960	82	695 230	79%	9460	71	10 600	84	23 160	79
Asian	229 400	8	80 540	8	5680	7	72 410	8	990	7	400	3	1050	4
Black	127 840	5	57 000	6	4120	5	48 810	6	1250	9	620	5	2210	8
Mixed	108 230	4	40 600	4	3070	4	33 770	4	1030	8	650	5	2080	7
Other	43 290	2	14 540	1	920	1	13 000	1	270	2	100	1	260	1
Missing	39 960	1	15 950	2	1680	2	13 190	2	250	2	280	2	560	2
First language														
English	2 452 530	87	881 940	87	74 060	88	758 650	87	11 160	84	11 180	88	26 900	92
Other	337 410	12	125 370	12	7750	9	112 980	13	1980	15	550	4	2130	7
Missing	17 280	1	8730	1	2600	3	4770	1	120	1	940	7	300	1
Free school meal eligibility														
No	2 319 150	83	740 170	73	59 180	70	648 730	74	7900	60	8380	66	15 960	54
Yes	488 050	17	275 850	27	25 220	30	227 660	26	5350	40	4270	34	13 360	46
Missing	40	k	30	k	10	k	10	k	c	–	c	–	c	–
IDACI quintile														
Q1: most deprived	669 230	24	318 550	31	24 170	29	274 720	31	4950	37	3310	26	11 410	39
Q2	565 980	20	231 480	23	18 670	22	199 590	23	3310	25	2700	21	7200	25
Q3	528 330	19	181 610	18	14 880	18	157 520	18	2210	17	2240	18	4770	16
Q4	520 120	19	151 740	15	12 830	15	132 090	15	1580	12	1850	15	3380	12
Q5: least deprived	510 840	18	125 690	12	11 240	13	109 550	13	1100	8	1550	12	2250	8
Missing	12 740	k	6970	1	2620	3	2930	k	100	1	1010	8	320	1
Chronic conditions														
No	2 383 760	85	810 510	80	48 280	57	720 740	82	11 470	87	6200	49	23 820	81
Yes	423 470	15	205 530	20	36 130	43	155 650	18	1780	13	6460	51	5500	19
Died	5800	k	3390	k	880	1	1970	k	80	1	310	2	150	1

Data are presented in accordance with the statistical control policy for linked CLA data such that national figures are rounded to the nearest 10 and percentages are rounded to 0 decimal places.

‘c’ in this table indicates that the figures have been suppressed to protect confidentiality, and ‘k’ is used when a result that is not zero would appear as zero due to rounding.

CLA, child looked after; EHCP, education, health and care plan; IDACI, Income Deprivation Affecting Children Index; SEN, special educational needs.

### Outcomes

The primary outcomes were planned and unplanned hospital admission rates at ages 11–23 year between 1 September 2007 and 1 March 2020.

Hospital admissions were defined as continuous time as an inpatient under the care of the National Health Service; therefore, consecutive admissions within 1 day of discharge (eg, hospital transfers) were treated as part of the same admission. We defined admission type (planned/unplanned) according to the admission method recorded at the start of admission.

Admissions related to pregnancy^10^ were expected to increase with age and were excluded from the primary outcomes but are reported as a secondary outcome for girls. This only captures information about pregnancies that resulted in delivery in a hospital; therefore, early pregnancy and loss are not likely to be captured.

Unplanned mental health-related admissions were investigated as a secondary outcome and reflect diagnostic codes for:

- Established and/or enduring mental health diagnoses defined using F chapter ICD-10 codes (mental, behavioural and neurodevelopmental disorders) recorded as any diagnosis.
- Injuries relating to drug and alcohol misuse, maltreatment, violence and intentional self-harm (including self-poisoning and self-cutting), recorded in any diagnostic position.
- Potential manifestations of stress, indicative of undifferentiated and/or transient symptoms recorded as the primary diagnosis, including medically unexplained abdominal pain. Specific medical exclusions (eg, appendectomy) recorded as part of the same continuous inpatient stay were applied.

We used all ICD-10 codes outlined in the ECHILD Code Repository: https://code.echild.ac.uk/srp_nichobhthaigh_v2. In this paper, we refer to these as mental health-related admissions (rather than stress-related presentations) to better communicate the mental health needs of young people with intersecting social and developmental challenges.

### Statistical analysis

We described the overall numbers and proportions of children and young people by pupil characteristic and across the six statutory support groups. To comply with statistical disclosure rules, all counts are rounded to the nearest 10; thus, counts may not sum to the total study population.

We calculated hospital admission rates (planned, unplanned and unplanned mental health-related admissions) per 100 person-years (100PY) at risk, which was calculated as the time from 1 September of Year 7 to the end of the follow-up, excluding admitted time in hospital (including pregnancy-related admissions). Point estimates are reported with associated 95% CIs. For rates stratified by age, we used the number of admissions and total person-time at risk during the school year corresponding to a given age (eg, for age 11 years, this would span from 1 September of Year 7 to 31 August). Rates stratified by age were derived as the total number of admissions divided by the total number of PY in a given year (aggregated over all pupils).

Crude hospital admission rate ratios (RR) and 95% CI were estimated for pupils with different levels of statutory support compared with peers with no support using negative binomial regression (due to overdispersion). Models were stratified by gender and age (categorised as 11–12, 13–15, 16–17 and 18–23 years; we derived the total number of admissions and PY at risk per pupil within each of these categories) and adjusted for year of birth (only) to account for potential cohort effects. We present stratified crude and minimally adjusted RRs in line with the study’s descriptive aims to quantify hospital admission rates among groups of pupils.

Data were analysed with R Studio and Stata V.18.^27 28^

### Public involvement

This study and the ECHILD database were informed by public contributors, including the Great Ormond Street Hospital National Children’s Bureau Families Research Advisory Group (see https://www.echild.ac.uk/engagements), who informed the selection of the research topic and definitions/terminology.

## Results

### Cohort characteristics

The cohort included 2 807 230 pupils: 1 368 120 girls (49%) and 1 439 110 (51%) boys. Overall, 64% (1 791 190) of pupils received no support, and 36% (n=1 016 040) received statutory support: 31% (n=876 400) had SEN support only, 3% (n=84 410) had EHCP only and <1% were care-experienced only (n=13 260), had an EHCP and were care-experienced (n=12 660) or had SEN support and care experience (n=29 330) (table 1). Boys were over-represented in those with any SEN provision, while girls were over-represented in the care-experienced only group (online supplemental appendix table 1). Of the 55 240 pupils who were care-experienced, 53% (29 330) had recorded SEN support and 23% (12 660) had an EHCP, such that 76% (41 990) had any recorded SEN provision (online supplemental appendix table 1).

Pupils receiving any statutory services were more likely to be eligible for free school meals (particularly care-experienced pupils), living in the most deprived 20% of areas and to have chronic conditions (particularly EHCP groups) compared with their peers without support. Between 1% and 2% of pupils who had an EHCP and/or were care-experienced died during the study period compared with <1% of peers with no support (table 1).

### Hospital admission rates

The proportion of pupils with at least one hospital admission by exposure group and gender is outlined in online supplemental appendix table 2 and visualised in online supplemental appendix figure 3.

**Figure 1:**
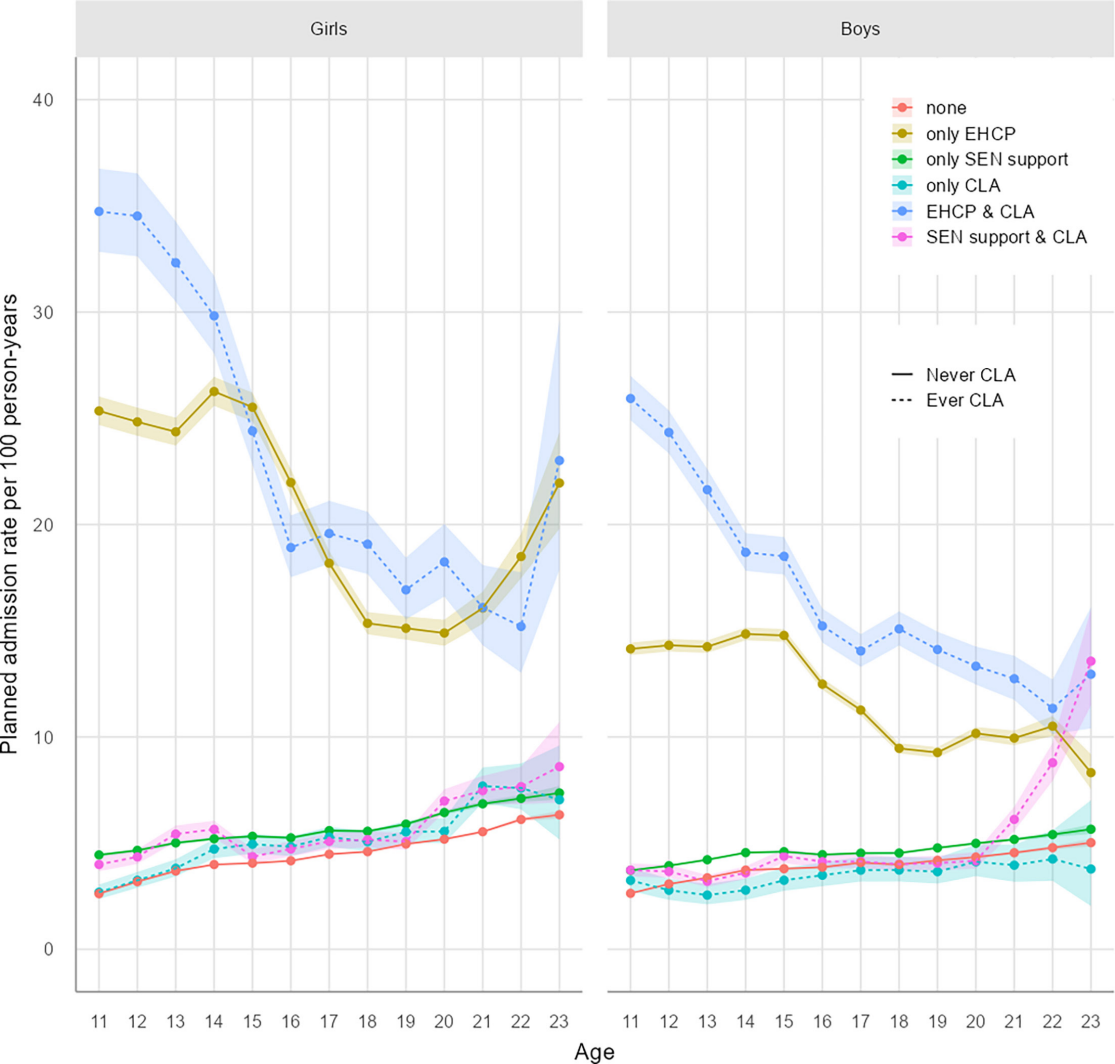
Planned hospital admission rates by age and exposure. CLA, children looked after; EHCP, education, health and care plan; SEN, special educational needs.

**Figure 2:**
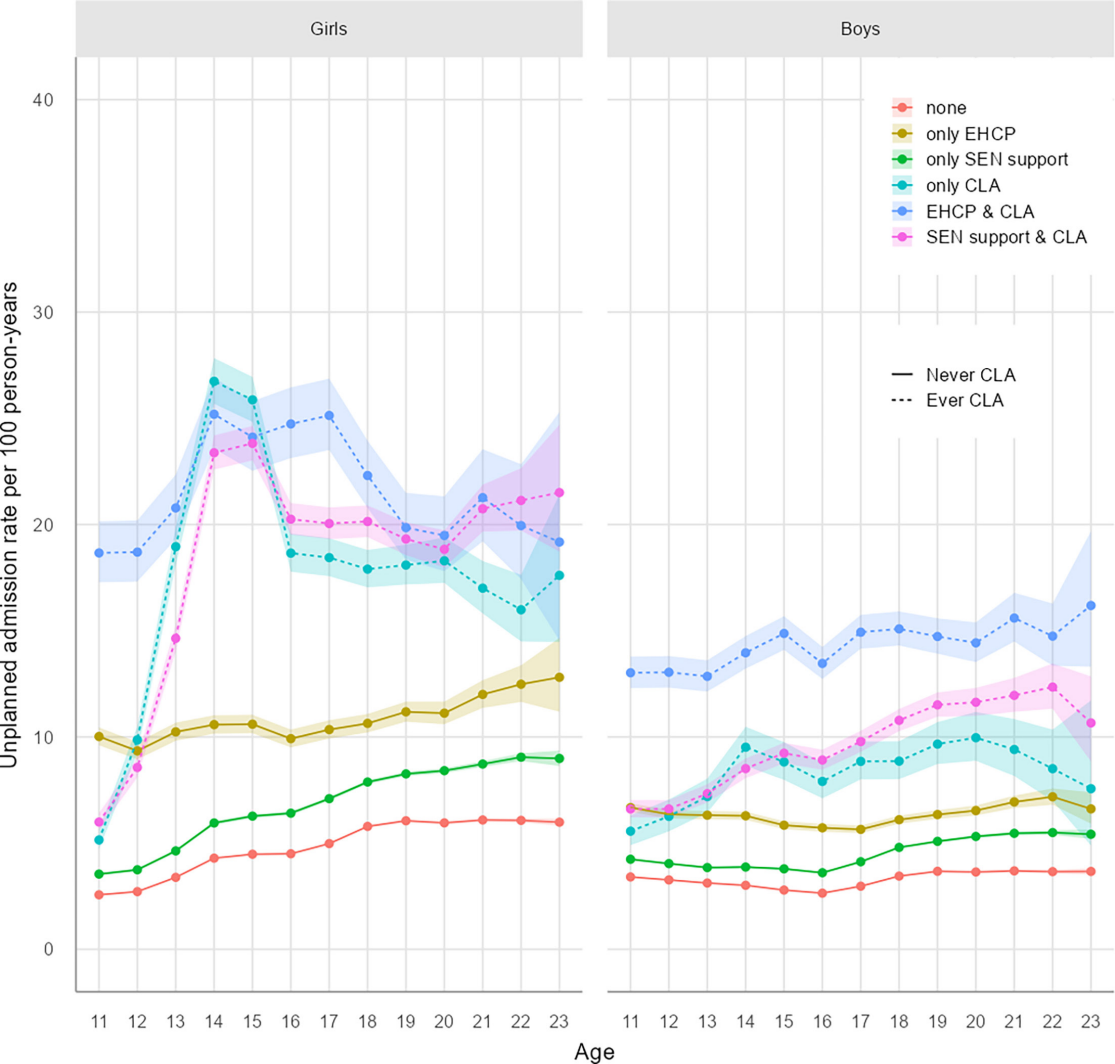
Unplanned hospital admission rates by age and exposure. CLA, children looked after; EHCP, education, health and care plan; SEN, special educational needs.

**Figure 3:**
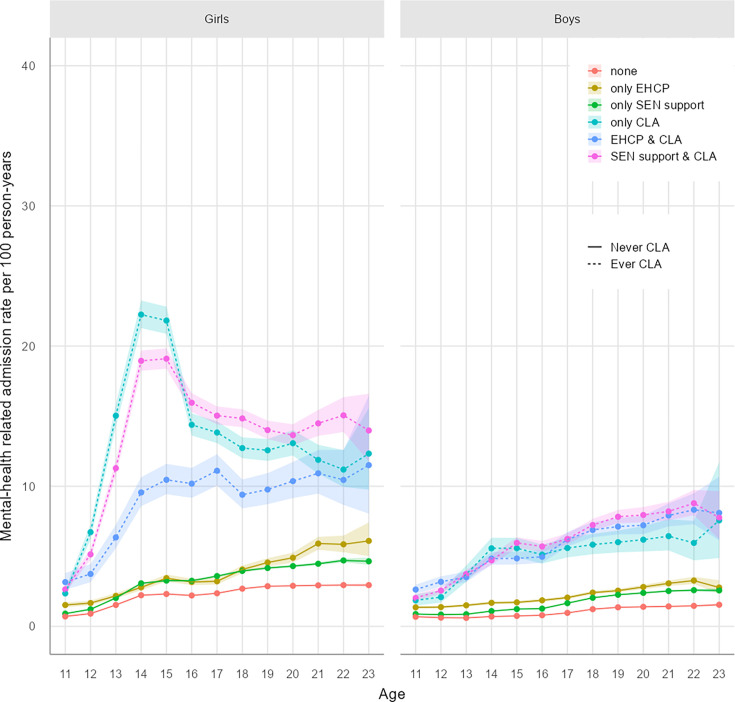
Mental health-related hospital admission rates by age and exposure. CLA, children looked after; EHCP, education, health and care plan; SEN, special educational needs.

Crude rates of hospital admissions are visualised in figure 1 (planned admissions), figure 2 (unplanned admissions), figure 3 (mental health-related admissions) and online supplemental appendix figure 2 (pregnancy-related admissions). The associated crude rates and 95% CIs for these figures are outlined in online supplemental appendix tables 3A (girls) and 3B (boys), respectively.

RRs for rates of admissions for each exposure group, relative to pupils with no support, are outlined in table 2 and visualised in online supplemental appendix figure 4 (4A, planned; 4B, unplanned; 4C, mental health-related and 4D, pregnancy-related admissions).

**Table 2:** RR for planned, unplanned, unplanned mental-health related and pregnancy-related hospital admissions by age and exposure group

Level of support	Girls				Boys			
	11–12 years	13–15 years	16–17 years	18+ years	11–12 years	13–15 years	16–17 years	18+ years
	RR for planned hospital admissions (relative to pupils with no support)							
EHCP only	8.67 (8.60 to 8.75)	6.64 (6.59 to 6.68)	4.66 (4.62 to 4.69)	2.86 (2.84 to 2.87)	4.79 (4.77 to 4.81)	3.77 (3.76 to 3.79)	2.74 (2.73 to 2.75)	1.90 (1.89 to 1.9)
SEN only	1.44 (1.44 to 1.44)	1.25 (1.24 to 1.25)	1.19 (1.19 to 1.19)	1.17 (1.17 to 1.18)	1.29 (1.29 to 1.29)	1.15 (1.15 to 1.15)	1.06 (1.05 to 1.06)	1.07 (1.07 to 1.07)
CLA only	1.04 (1.01 to 1.06)	1.19 (1.17 to 1.21)	1.12 (1.09 to 1.14)	1.15 (1.13 to 1.16)	1.10 (1.04 to 1.16)	0.82 (0.79 to 0.86)	1.00 (0.95 to 1.04)	0.81 (0.78 to 0.84)
EHCP and CLA	14.1 (13.8 to 14.5)	8.21 (8.04 to 8.39)	4.88 (4.73 to 5.03)	3.58 (3.5 to 3.67)	10.3 (10.2 to 10.5)	6.32 (6.25 to 6.4)	4.57 (4.51 to 4.64)	3.87 (3.83 to 3.91)
SEN and CLA	1.26 (1.24 to 1.28)	1.12 (1.11 to 1.14)	1.10 (1.08 to 1.12)	1.15 (1.14 to 1.16)	1.26 (1.24 to 1.28)	1.07 (1.06 to 1.09)	1.03 (1.02 to 1.05)	0.96 (0.95 to 0.97)
	RR for unplanned hospital admissions (relative to pupils with no support)							
EHCP only	3.37 (3.34 to 3.4)	2.42 (2.4 to 2.44)	2.09 (2.07 to 2.11)	1.84 (1.83 to 1.85)	1.7 (1.69 to 1.7)	1.77 (1.77 to 1.78)	1.81 (1.8 to 1.81)	1.65 (1.65 to 1.66)
SEN only	1.33 (1.33 to 1.33)	1.39 (1.38 to 1.39)	1.44 (1.44 to 1.45)	1.42 (1.42 to 1.42)	1.23 (1.23 to 1.23)	1.29 (1.29 to 1.29)	1.39 (1.39 to 1.39)	1.44 (1.44 to 1.44)
CLA only	2.91 (2.87 to 2.96)	6.24 (6.19 to 6.3)	4.06 (4.02 to 4.11)	3.06 (3.04 to 3.09)	1.88 (1.81 to 1.95)	3.29 (3.21 to 3.36)	3.33 (3.24 to 3.43)	2.87 (2.81 to 2.93)
EHCP and CLA	7.59 (7.36 to 7.83)	5.57 (5.44 to 5.7)	5.05 (4.91 to 5.19)	3.34 (3.27 to 3.42)	4.04 (3.98 to 4.1)	4.92 (4.86 to 4.98)	5.28 (5.21 to 5.36)	4.23 (4.18 to 4.27)
SEN and CLA	2.77 (2.74 to 2.81)	5.29 (5.25 to 5.33)	4.56 (4.52 to 4.6)	3.47 (3.45 to 3.49)	1.95 (1.93 to 1.98)	2.84 (2.81 to 2.86)	3.38 (3.35 to 3.41)	3.08 (3.06 to 3.1)
	RR for mental health-related hospital admissions (relative to pupils with no support)							
EHCP only	1.92 (1.88 to 1.97)	1.38 (1.36 to 1.4)	1.43 (1.41 to 1.45)	1.72 (1.71 to 1.74)	1.94 (1.93 to 1.96)	2.3 (2.29 to 2.31)	2.27 (2.26 to 2.29)	2.01 (2 to 2.02)
SEN only	1.31 (1.31 to 1.31)	1.41 (1.41 to 1.42)	1.56 (1.56 to 1.56)	1.54 (1.54 to 1.55)	1.31 (1.31 to 1.31)	1.63 (1.63 to 1.63)	1.78 (1.78 to 1.78)	1.77 (1.77 to 1.77)
CLA only	6.21 (6.09 to 6.32)	10.4 (10.3 to 10.5)	6.46 (6.38 to 6.53)	4.53 (4.49 to 4.57)	3.43 (3.24 to 3.64)	9.09 (8.82 to 9.36)	7.51 (7.25 to 7.78)	5.12 (4.99 to 5.26)
EHCP and CLA	4.45 (4.15 to 4.76)	3.67 (3.53 to 3.82)	4.11 (3.94 to 4.29)	2.9 (2.81 to 3)	4.47 (4.33 to 4.62)	6.81 (6.66 to 6.96)	6.79 (6.63 to 6.95)	5.36 (5.28 to 5.45)
SEN and CLA	5.02 (4.94 to 5.1)	8.49 (8.42 to 8.55)	7.44 (7.38 to 7.51)	5.43 (5.39 to 5.46)	3.71 (3.64 to 3.78)	7.6 (7.52 to 7.69)	7.63 (7.54 to 7.72)	5.87 (5.82 to 5.92)
	RR for pregnancy-related hospital admissions (relative to pupils with no support)							
EHCP only	N/A	0.96 (0.93 to 0.99)	1.31 (1.28 to 1.33)	1.13 (1.12 to 1.14)				
SEN only		2.22 (2.21 to 2.23)	2.62 (2.61 to 2.63)	2.19 (2.18 to 2.19)				
CLA only		11.4 (11.1 to 11.7)	8.31 (8.17 to 8.45)	4.54 (4.49 to 4.59)				
EHCP and CLA		1.20 (1.04 to 1.38)	1.53 (1.43 to 1.64)	1.28 (1.23 to 1.32)				
SEN and CLA		10.1 (9.9 to 10.29)	8.28 (8.19 to 8.39)	5.25 (5.21 to 5.30)				

Pupils with no support are the baseline group (RR of 1) for all RRs.

CLA, children looked after; EHCP, education, health and care plan; RR, rate ratio; SEN, special educational needs.

#### Planned admission rates

More than 1 in 5 of all pupils had at least one planned admission, with significant variation by age and level of support (online supplemental appendix table 2; online supplemental appendix figure 3A). Overall, girls had 4.83 planned admissions per 100PY, and boys had 4.50/100PY. Rates were higher for girls than for boys across all ages and exposure/support groups (figure 1).

Planned admission rates were consistently higher across all ages for boys and girls receiving any kind of statutory support than peers with no support, except for some care-experienced boys without an EHCP/SEN (figure 1; table 2 and online supplemental appendix figure 3A). For these care-experienced boys, planned admission rates were 18%–19% lower at ages 13–15 years (RR 0.82 (0.79 to 0.86)) and 18+ years (RR 0.81 (0.78 to 0.84)) than for peers of the same age with no statutory support (figure 1; online supplemental appendix figure 4A). Rates were highest for pupils of secondary school ages (11–15 years old) with EHCPs, especially 11–12-year-old girls (RR 14.1 (13.8 to 14.5)) and boys (RR 10.3 (10.2 to 10.5)) who were also care-experienced (figure 1; table 2 and online supplemental appendix figure 4A). Relative differences in planned admission rates for children with an EHCP compared with peers with no support reduced with age (online supplemental appendix figure 4A). This reflects a rapid decline in planned admissions among EHCP groups after secondary school age, coinciding with the transition into adult healthcare services (figure 1). Conversely, among pupils with no support and for pupils in the SEN/CLA/both groups, planned admission rates increased with increasing age (figure 1), although we caution that the number of events and follow-up time for the oldest adolescents in this study (21–23 years) is relatively small compared with younger ages.

#### Unplanned admission rates

One in four boys and girls had at least one unplanned hospital admission, with significant variation by age and level of support (online supplemental appendix table 2/figure 3B). Overall, girls had higher unplanned admission rates than boys (5.35/100PY (5.34 to 5.36) vs 3.92/100PY (3.91 to 3.93)) (figure 2 and online supplemental appendix tables 3A and 3B).

Unplanned admission rates were higher among all groups with statutory support than peers with no support (table 2 and online supplemental appendix figure 4B; all RRs have 95% CI>1) and highest in care-experienced girls who had an EHCP (21.9 (95% CI 21.4 to 22.4)/100PY). Similar, but generally higher, trends in unplanned admission rates were observed for pupils receiving SEN support (only), and these differences widened with age (table 2). Patterning of admission rates by age was particularly striking for girls (with a strong peak, aged 14–15 years; figure 2), and especially for care-experienced groups where rates were over five times higher relative to peers with no support (table 2, RR for girls aged 13–15 years: care-experienced only, 6.24 (6.19 to 6.30); EHCP and care-experienced, 5.57 (5.44 to 5.7) and SEN and care-experience, 5.29 (5.25 to 5.33)). Care-experienced boys had 2–5 times higher rates of unplanned admissions than peers with no support, and these differences were largest at ages 16–17 years (table 2, RR for boys aged 16–17 years: care-experienced only, 3.33 (3.24 to 3.42); EHCP and care-experienced, 5.28 (5.21 to 5.36) and SEN and care-experienced, 3.38 (3.35 to 3.41)).

#### Mental health-related admissions

Overall, 14% of girls and 9% of boys had at least one mental health-related admission, with significant variation by age and group (online supplemental appendix table 2 and online supplemental appendix figure 3C). Girls had higher overall rates of mental health-related admissions than boys (2.56/100PY (2.55 to 2.57) vs 1.28/100PY (1.28 to 1.29); figure 3 and online supplemental appendix tables 3A and 3B).

For both boys and girls, there were strong similarities in the patterning of mental health-related admissions for groups with and without care experience. For care-experienced girls, patterns of unplanned mental health-related admissions were similar to those for all unplanned admissions, peaking around ages 14–15 years. For care-experienced boys, mental health-related admissions more consistently increased with age (figure 3). Overall, 48% of all unplanned admissions for girls and 33% for boys were mental health-related. Rates of mental health-related admissions for pupils with no support were 2.11/100PY (2.10 to 2.12) for girls and 0.94/100PY (0.93 to 0.94) for boys, which accounts for 46% and 29% of all unplanned admissions, respectively (online supplemental appendix tables 3A and 3B). Mental health-related unplanned admission rates were highest for care-experienced girls (CLA only, 13.3/100PY (13.1 to 13.6); CLA and SEN, 13.2 (13.0 to 13.3) and CLA and EHCP, 8.53 (8.23 to 8.83)) and boys (CLA only, 4.79/100PY (4.59 to 5.00); CLA and SEN, 5.51 (5.39 to 5.63) and CLA and EHCP, 5.28 (5.14 to 5.43)) (figure 3 and online supplemental appendix tables 3A and 3B). These mental health-related admissions accounted for three-quarters of all unplanned admissions in care-experienced girls without an EHCP (CLA only, 75% and CLA and SEN, 74%) and for 39% of care-experienced girls with an EHCP. In care-experienced boys, mental health-related admissions accounted for over half of unplanned admissions in those without an EHCP (CLA only, 58% and CLA and SEN, 60%) and for 37% of care-experienced boys with an EHCP. In contrast to rates of all unplanned admissions, it was notable that care-experienced young people with an EHCP had lower rates of mental health-related admissions than SEN and care-experienced groups, particularly for girls.

#### Pregnancy-related admissions

Overall, 14% of girls (4.0/100PY (3.99 to 4.01)) (online supplemental appendix table 2 and online supplemental appendix table 3A) had at least one pregnancy-related admission: rates increased with age (online supplemental appendix figure 2).

Among care-experienced (only) girls, 39% (14.5/100PY (14.3 to 14.8)) had a pregnancy-related admission. Compared with girls with no support, pregnancy-related admission rates were raised in all care-experienced groups and were relatively highest among 13–15-year-olds without an EHCP (RR aged 13–15 years: care-experienced only, 11.4 (11.1 to 11.7) and SEN and care experience, 10.1 (9.9 to 10.3); table 2 and online supplemental appendix figure 4D). Rates were twofold higher (table 2) for girls with SEN support (only) compared with peers with no support (6.74/100PY (6.71 to 6.77) vs 2.96/100PY (2.95 to 2.97)).

## Discussion

This study takes a multi-sectoral approach to quantify the first whole-nation estimates of hospital admissions for pupils in England receiving statutory support. Overall, more than 1 in 5 adolescents in England experienced planned and unplanned hospital admissions, and unplanned admission rates were higher among all pupil groups with SEN and/or experience of care (vs pupils with no support). In contrast, rates of planned admissions were substantially higher for pupils with an EHCP (reflecting medical criteria for EHCP eligibility), but not in other groups receiving support, which suggests potential unmet need.

Unplanned admission rates were particularly high for care-experienced pupils, especially for those with coexisting SEN provision. Among care-experienced pupils, high proportions (half for boys and two-thirds for girls) of unplanned admissions were mental health-related, indicating urgent unmet mental health needs. For care-experienced pupils with an EHCP (particularly girls), mental health-related admissions accounted for a lower proportion of unplanned admissions than for care-experienced pupils without an EHCP. This may reflect underlying differences in medical complexity, independence and risk-taking behaviours, as well as recognition of mental health-related presentations. Rates of planned hospital admissions for care-experienced groups and groups with no support were similar, except for care-experienced boys aged 13–15 years and 18+ years, where rates were one-fifth lower. This is important because care-experienced pupils had higher rates of unplanned admissions, particularly for mental health. For girls, pregnancy-related admissions were higher for all groups receiving support, except for girls with an EHCP (only). Over 1 in 3 care-experienced girls (vs 1 in 9 girls with no support) had a pregnancy-related admission during adolescence, and rates were over 10-fold higher among 13–15-year-olds, highlighting the sexual vulnerability of this group.

This study differentiates between groups of pupils receiving SEN support and/or care experience to reflect differences in medical and social factors; for example, greater medical needs among pupils with an EHCP and greater social adversity among care-experienced children and young people. We report higher unplanned admission rates among girls (peaking at ages 14–15 years), which mirrors other studies and may reflect biopsychosocial and structural factors (eg, prioritising admissions for under 16s, particularly suspected self-harm) that warrant further mixed-methods research.^12 13 29^

Our results suggest that care-experienced pupils, particularly boys, have similar or lower rates of planned care than pupils with no support. Analyses for Scotland report a higher prevalence of chronic health conditions and mental health admissions for care-experienced young people than the general population,^30^ indicating that our findings likely reflect unmet preventative care needs. Similarly, raised unplanned admission rates have been reported for young people in Wales with social care contact.^31^ Care-experienced young people often experience additional barriers to their peers, including inconsistent carer support for healthcare seeking and access, and face particular challenges accessing mental healthcare.^29 30^ High rates of mental health-related admissions to acute hospital wards do not reflect best practice for managing mental health and indicate that community and specialist services are not meeting demand.^31^,^33^ We report high rates of pregnancy-related admissions for care-experienced girls, mirroring findings for Scotland and raising concerns over sexual exploitation and safeguarding, as well as a need for intensive interventions such as the Family Nurse Partnership to limit intergenerational transmission of disadvantage.^3 34 35^

Strengths of the study include using longitudinal, multi-sectoral linked administrative datasets to examine health inequalities across statutory support groups and whole-country coverage to minimise selection bias.^36^ These groupings reflect pupils known to child health services, with actionable messages for social care and health. Limitations reflect variations in real-world testing and support for SEN and/or care experience, such that need is imperfectly captured and actual provision is unmeasured. Although we applied a relatively broad time window in which to measure care experience, the lack of linkable data for those under 5 years means that the early life experiences of care are likely under-ascertained in this study.

The broad range of adolescent ages (11–23 years) is a strength of the study. However, adolescents over the age of 20 years are under-represented relative to younger individuals, meaning that results for the oldest adolescents in the cohort are less precise and may generalise less well relative to results for younger groups. Young people who are not enrolled in state-funded education for reasons reflecting school or child/family circumstances (eg, pushing out/off-rolling, independent education or homeschooling) are an important group, have needs that are likely under-ascertained in this study and should be the focus of future research.^37^ We applied a broader case definition for mental health-related admissions, notably including potentially psychosomatic presentations, that captures proportionately more admissions than other studies (eg, 28% of admissions in 2021–2022 for girls aged 11–15 years and 6% for boys^12^). These differences reflect the relatively high frequency of psychosomatic presentations relative to other mental health-related presentations, particularly among minoritised groups of adolescents and boys.^13 38^ Further investigation of pathways across services, including adolescent mental health services, and with additional linkage to adult service data, is needed to better understand the specificity of this definition.^39^ Finally, our study does not reflect the pandemic or post-pandemic periods, and outcomes may have worsened following the erosion of youth services, likely with disproportionate impacts for young people in care.^40 41^

Our findings emphasise both greater healthcare needs for young people with additional educational and/or social care needs (relative to peers) and imbalances in planned and unplanned hospital admissions. Action is most urgently needed for care-experienced young people and those who are in their mid-teens or ageing out of children’s services. For these care-experienced groups, rates of mental health-related admissions and teenage pregnancy are much higher than those of their peers, indicating that these young people do not receive appropriate support. Continued tailored support into post-16 education and social care must be young-person-centred, trauma-informed and integrated across services. Finally, our results indicate a need for enhanced co-ordination of services spanning social care, education and health for young people with additional needs to ensure that care is seamless and effective.

## Supplementary material

10.1136/bmjph-2025-003106 online supplemental file 1

## Data Availability

Data may be obtained from a third party and are not publicly available.
