# Supplementary material for: Hospital admissions among adolescents with local authority care experience or special educational needs in England: a population-based cohort study using linked administrative data from health, education and social care services
Source: BMJ Public Health. 2026 Mar 12;4(1):e003106. doi: 10.1136/bmjph-2025-003106 (PMC12983678; doi:10.1136/bmjph-2025-003106)
Supplement: online supplemental file 1 [file bmjph-4-1-s001.pdf]

Supplementary materials

Appendix Figure 1 - Cohort overview

Academic year of data (1st Sept - 31st Aug)

Hospital Episode Statistics (HES) Admitted Patient Care (APC)

a Office for National Statistics (ONS) mortality records

National Pupil Database (NPD) School Census Pupil Level

b c Children Looked After (CLA) Return

Academic year of birth (1st Sept - 31st August)	1997/98	1998/99	1999/00	2000/01	2001/02	2002/03	2003/04	2004/05	2005/06	2006/07	**2007/08**	**2008/09**	**2009/10**	**2010/11**	**2011/12**	2012/13	2013/14	2014/15	2015/16	2016/17	2017/18	2018/19	2019/20**
**1996/97**	1	2	3	4 (R)	5 (Y1)	6 (Y2)	7 (Y3)	8 (Y4)	9 (Y5)	10 (Y6)	**11 (Y7)**	12 (Y8)	13 (Y9)	14 (Y10)	15 (Y11)	16	17	18	19	20	21	22	23
**1997/98**	0	1	2	3	4 (R)	5 (Y1)	6 (Y2)	7 (Y3)	8 (Y4)	9 (Y5)	10 (Y6)	**11 (Y7)**	12 (Y8)	13 (Y9)	14 (Y10)	15 (Y11)	16	17	18	19	20	21	22
**1998/99**		0	1	2	3	4 (R)	5 (Y1)	6 (Y2)	7 (Y3)	8 (Y4)	9 (Y5)	10 (Y6)	**11 (Y7)**	12 (Y8)	13 (Y9)	14 (Y10)	15 (Y11)	16	17	18	19	20	21
**1999/00**			0	1	2	3	4 (R)	5 (Y1)	6 (Y2)	7 (Y3)	8 (Y4)	9 (Y5)	10 (Y6)	**11 (Y7)**	12 (Y8)	13 (Y9)	14 (Y10)	15 (Y11)	16	17	18	19	20
**2000/01**				0	1	2	3	4 (R)	5 (Y1)	6 (Y2)	7 (Y3)	8 (Y4)	9 (Y5)	10 (Y6)	**11 (Y7)**	12 (Y8)	13 (Y9)	14 (Y10)	15 (Y11)	16	17	18	19

Primary school

Secondary school

a: Partial coverage of an academic year as ONS Mortality data were first linked to HES in January 1998.

b: Partial coverage of population as between 01/04/1992 & 31/03/2003, CLA data were only collected for a one-third sample (i.e., children with a day of birth divisible by 3).

c: Partial coverage as linkage between NPD and CLA began on 1 April 2005 for CLA

***follow-up until 1st March 2020*

Appendix Table 1 – distribution of boys and girls within each of the two-level CLA and three-level SEN exposure groups

	**Females**				**Males**			
**Care Experience**	**No SEN provision**	**SEN support**	**EHCP**	**Total**	**No SEN provision**	**SEN support**	**EHCP**	**Total**
**No**	985,080 (72%)	334,880 (24%)	21,710 (2%)	1,341,660 (98%)	806,110 (56%)	541,520 (38%)	62,700 (4%)	1,410,330 (98%)
**Yes: CLA**	8,940 (1%)	14,030 (1%)	3,500 (*k*%)	26,460 (2%)	4,320 (*k*%)	15,300 (1%)	9,160 (1%)	28,780 (2%)
**Total**	994,010 (73%)	348,900 (26%)	25,210 (2%)	1,368,120 (100%)	810,430 (56%)	556,820 (39%)	71,870 (5%)	1,439,110 (100%)

CLA: child looked after; EHCP: education, health and care plan; SEN: Special Educational Needs. Data are presented in accordance with statistical control policy for linked CLA data such that national figures are rounded to the nearest 10 and percentages are rounded to 0 decimal places 'k' is used when a result that is not zero would appear as zero due to rounding.

Appendix Figure 2: Pregnancy-related hospital admission rates by age and exposure

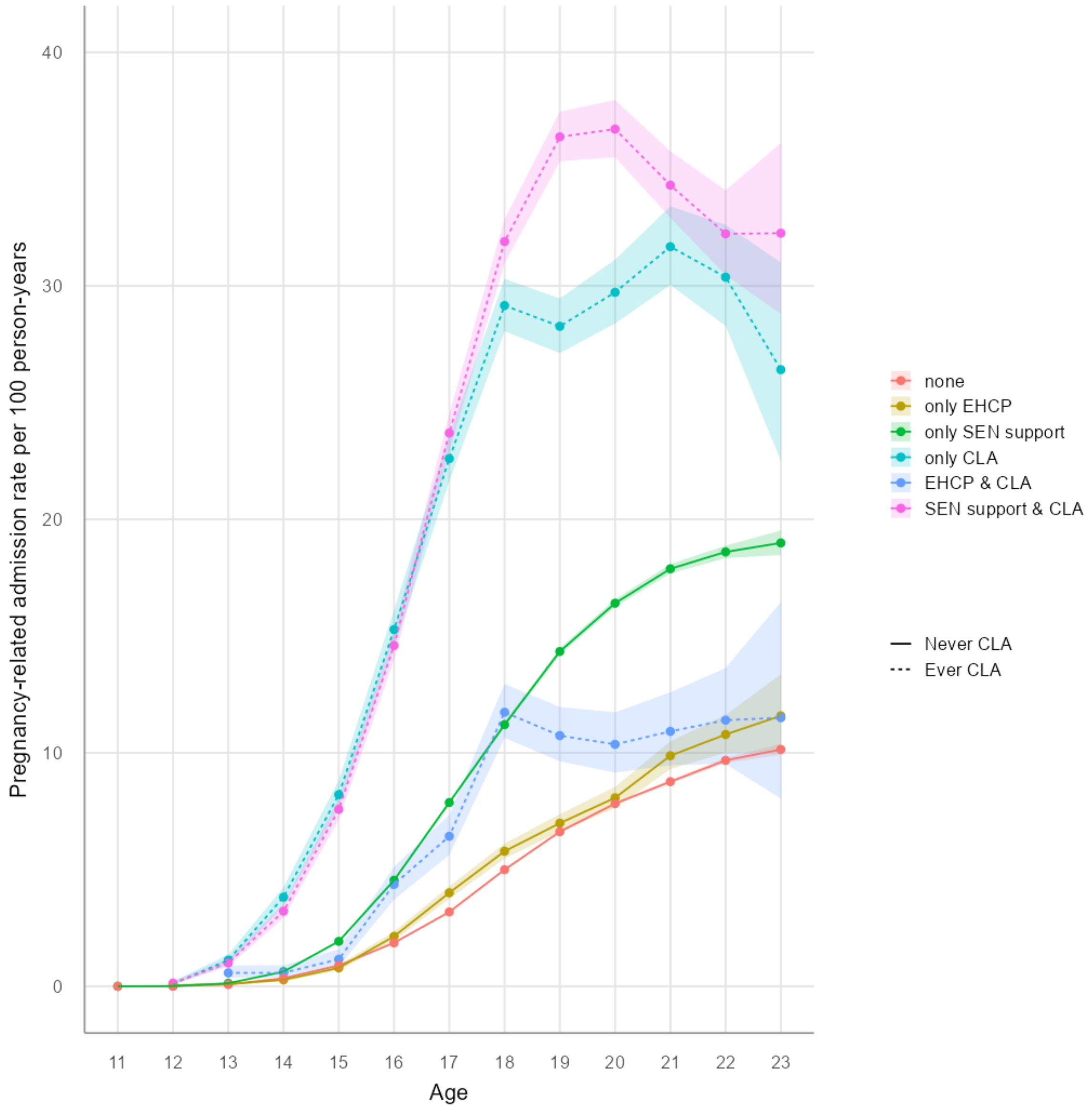

CLA: children looked after, EHCP: education, health and care plan; SEN: Special Educational Needs

Appendix Table 2 – proportion of pupils with at least one hospital admission during the follow-up period by exposure group and gender

		Planned	Unplanned	Mental-health related	Pregnancy-related	Any admission
Girls	Total	316,020 (23%)	357,030 (26%)	196,710 (14%)	195,540 (14%)	621,320 (45%)
	No support	217,650 (22%)	238,240 (24%)	126,720 (13%)	112,130 (11%)	416,420 (42%)
	SEN support only	82,130 (25%)	98,210 (29%)	56,310 (17%)	71,160 (21%)	173,460 (52%)
	EHCP only	8,860 (41%)	7,320 (34%)	3,460 (16%)	2,200 (10%)	12,620 (58%)
	CLA only	2,220 (25%)	4,550 (51%)	3,690 (41%)	3,530 (39%)	6,290 (70%)
	SEN support & CLA	3,580 (26%)	7,050 (50%)	5,640 (40%)	6,010 (43%)	10,100 (72%)
	EHCP & CLA	1,600 (46%)	1,670 (48%)	890 (25%)	520 (15%)	2,440 (70%)
Boys	Total	307,370 (21%)	349,590 (24%)	133,580 (9%)		533,430 (37%)
	No support	163,190 (20%)	177,550 (22%)	60,370 (7%)		280,260 (35%)
	SEN support only	117,190 (22%)	142,530 (26%)	58,080 (11%)		209,670 (39%)
	EHCP only	19,250 (31%)	17,700 (28%)	7,790 (12%)		28,410 (45%)
	CLA only	860 (20%)	1,670 (39%)	1,040 (24%)		2,030 (47%)
	SEN support & CLA	3,300 (22%)	6,240 (41%)	4,100 (27%)		7,630 (50%)
	EHCP & CLA	3,590 (39%)	3,900 (43%)	2,190 (24%)		5,440 (59%)

CLA: child looked after; EHCP: education, health and care plan; SEN: Special Educational Needs. Data are presented in accordance with statistical control policy for linked CLA data such that national figures are rounded to the nearest 10 and percentages are rounded to 0 decimal places 'k' is used when a result that is not zero would appear as zero due to rounding.

Appendix Figure 3 – Proportion of children with at least one planned/unplanned admission by age and exposure group

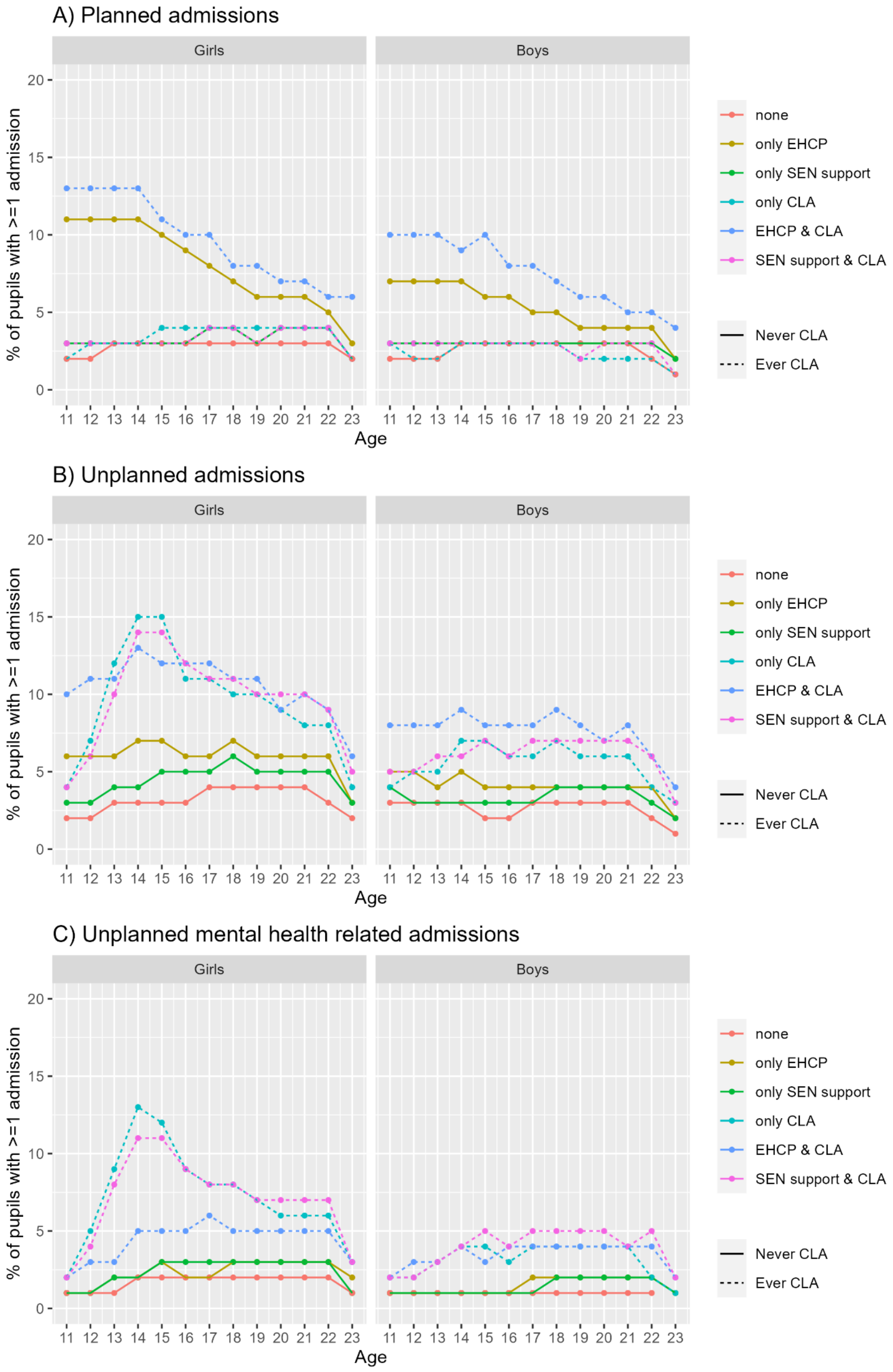

CLA: children looked after, EHCP: education, health and care plan; SEN: Special Educational Needs
all % were rounded to zero decimal places due to statistical disclosure rules

Appendix Table 3 A–Admission rates (per 100 PY) aged 11-23 years old by gender and level of statutory provision for girls

	Girls				
**Age**	11-12 years	13-15 years	16-17 years	18+ years	Total
**Planned rates**					
**Total**	3.75 (3.73, 3.78)	4.64 (4.62, 4.66)	4.89 (4.86, 4.91)	5.59 (5.57, 5.61)	4.83 (4.82, 4.84)
**No support**	2.90 (2.88, 2.93)	3.91 (3.89, 3.94)	4.32 (4.29, 4.35)	5.11 (5.09, 5.14)	4.20 (4.19, 4.21)
**SEN support only**	4.55 (4.50, 4.60)	5.18 (5.14, 5.22)	5.42 (5.36, 5.47)	6.18 (6.14, 6.23)	5.44 (5.41, 5.46)
**EHCP only**	25.09 (24.62, 25.56)	25.38 (24.99, 25.77)	20.08 (19.65, 20.50)	15.72 (15.43, 16.00)	21.11 (20.92, 21.30)
**CLA only**	2.97 (2.72, 3.22)	4.49 (4.24, 4.75)	5.06 (4.73, 5.39)	5.90 (5.63, 6.18)	4.77 (4.63, 4.91)
**SEN support & CLA**	4.21 (3.97, 4.45)	5.17 (4.95, 5.39)	4.94 (4.68, 5.20)	6.05 (5.83, 6.27)	5.23 (5.11, 5.34)
**EHCP & CLA**	34.63 (33.25, 36.02)	28.86 (27.82, 29.90)	19.25 (18.21, 20.30)	17.66 (16.91, 18.42)	24.44 (23.93, 24.95)
**Unplanned rates**					
**Total**	3.12 (3.10, 3.14)	4.88 (4.86, 4.91)	5.61 (5.59, 5.64)	6.87 (6.85, 6.89)	5.35 (5.34, 5.36)
**No support**	2.64 (2.62, 2.67)	4.05 (4.03, 4.08)	4.74 (4.71, 4.77)	5.96 (5.94, 5.99)	4.55 (4.54, 4.56)
**SEN support only**	3.64 (3.60, 3.69)	5.62 (5.57, 5.66)	6.75 (6.69, 6.81)	8.33 (8.28, 8.38)	6.36 (6.33, 6.38)
**EHCP only**	9.68 (9.39, 9.97)	10.47 (10.22, 10.72)	10.13 (9.83, 10.43)	11.28 (11.04, 11.52)	10.53 (10.39, 10.66)
**CLA only**	7.50 (7.11, 7.91)	23.85 (23.27, 24.44)	18.49 (17.86, 19.13)	17.70 (17.23, 18.18)	17.67 (17.40, 17.94)
**SEN support & CLA**	7.31 (7.00, 7.63)	20.61 (20.18, 21.04)	20.11 (19.59, 20.64)	19.85 (19.45, 20.25)	17.70 (17.48, 17.91)
**EHCP & CLA**	18.68 (17.67, 19.70)	23.36 (22.43, 24.29)	24.93 (23.75, 26.13)	20.76 (19.95, 21.58)	21.90 (21.41, 22.38)
**Mental health-related**					
**Total**	0.95 (0.93, 0.96)	2.50 (2.48, 2.51)	2.81 (2.79, 2.83)	3.40 (3.38, 3.42)	2.56 (2.55, 2.57)
**No support**	0.81 (0.80, 0.82)	2.02 (2.00, 2.04)	2.28 (2.26, 2.30)	2.84 (2.82, 2.86)	2.11 (2.10, 2.12)
**SEN support only**	1.06 (1.04, 1.09)	2.79 (2.75, 2.82)	3.42 (3.38, 3.47)	4.23 (4.20, 4.27)	3.06 (3.04, 3.08)
**EHCP only**	1.59 (1.48, 1.71)	2.80 (2.67, 2.93)	3.19 (3.02, 3.36)	4.84 (4.68, 5.00)	3.31 (3.24, 3.39)
**CLA only**	4.59 (4.28, 4.91)	19.69 (19.16, 20.23)	14.05 (13.50, 14.60)	12.50 (12.10, 12.89)	13.33 (13.10, 13.57)
**SEN support & CLA**	3.92 (3.69, 4.16)	16.42 (16.03, 16.80)	15.50 (15.04, 15.96)	14.33 (13.99, 14.67)	13.15 (12.96, 13.33)
**EHCP & CLA**	3.45 (3.02, 3.89)	8.78 (8.21, 9.36)	10.64 (9.89, 11.42)	10.04 (9.48, 10.61)	8.53 (8.23, 8.83)
**Pregnancy-related**					
**Total**		0.61 (0.61, 0.62)	3.72 (3.70, 3.74)	9.38 (9.35, 9.41)	4.00 (3.99, 4.01)

**No support**		0.44 (0.43, 0.45)	2.52 (2.50, 2.55)	7.05 (7.02, 7.08)	2.96 (2.95, 2.97)
**SEN support only**		0.90 (0.88, 0.91)	6.21 (6.15, 6.27)	14.83 (14.76, 14.90)	6.36 (6.33, 6.38)
**EHCP only**		0.39 (0.34, 0.43)	3.07 (2.91, 3.24)	7.72 (7.53, 7.92)	3.26 (3.19, 3.34)
**CLA only**		4.38 (4.13, 4.64)	18.94 (18.30, 19.58)	29.41 (28.80, 30.02)	14.52 (14.28, 14.77)
**SEN support & CLA**		3.93 (3.74, 4.12)	19.18 (18.67, 19.70)	34.41 (33.89, 34.95)	16.02 (15.82, 16.23)
**EHCP & CLA**		0.68 (0.52, 0.84)	5.39 (4.86, 5.95)	11.05 (10.46, 11.65)	4.90 (4.67, 5.13)

CLA: child looked after; EHCP: education, health and care plan; SEN: Special Educational Needs. Data are presented in accordance with statistical control policy for linked CLA data such that national figures are rounded to the nearest 10. Data on pregnancy-related admissions in girls aged 11-12 years are not presented due to small cell sizes.

Appendix Table 3 B–Admission rates (per 100 PY) aged 11-23 years old by gender and level of statutory provision for boys

	Boys				
Age	11-12 years	13-15 years	16-17 years	18+ years	Total
**Planned Rates**					
**Total**	3.87 (3.84, 3.89)	4.52 (4.50, 4.54)	4.58 (4.55, 4.60)	4.81 (4.79, 4.83)	4.50 (4.49, 4.51)
**No support**	2.86 (2.83, 2.88)	3.63 (3.61, 3.66)	3.97 (3.94, 4.00)	4.28 (4.26, 4.31)	3.77 (3.75, 3.78)
**SEN support only**	3.83 (3.79, 3.86)	4.46 (4.42, 4.49)	4.49 (4.45, 4.53)	4.88 (4.85, 4.91)	4.48 (4.47, 4.50)
**EHCP only**	14.23 (14.02, 14.44)	14.62 (14.45, 14.80)	11.87 (11.68, 12.06)	9.69 (9.56, 9.82)	12.39 (12.30, 12.47)
**CLA only**	3.01 (2.65, 3.38)	2.94 (2.65, 3.24)	3.61 (3.21, 4.01)	3.80 (3.49, 4.11)	3.38 (3.21, 3.56)
**SEN support & CLA**	3.70 (3.48, 3.91)	3.73 (3.55, 3.91)	4.13 (3.91, 4.36)	4.93 (4.74, 5.12)	4.19 (4.09, 4.29)
**EHCP & CLA**	25.13 (24.41, 25.86)	19.61 (19.09, 20.14)	14.69 (14.13, 15.25)	13.78 (13.37, 14.19)	17.81 (17.54, 18.08)
**Unplanned Rates**					
**Total**	3.88 (3.86, 3.90)	3.58 (3.56, 3.60)	3.49 (3.47, 3.51)	4.47 (4.45, 4.49)	3.92 (3.91, 3.93)
**No support**	3.34 (3.31, 3.37)	2.98 (2.95, 3.00)	2.81 (2.78, 2.83)	3.60 (3.58, 3.63)	3.22 (3.21, 3.23)
**SEN support only**	4.14 (4.10, 4.18)	3.84 (3.81, 3.87)	3.86 (3.83, 3.90)	5.15 (5.11, 5.18)	4.33 (4.32, 4.35)
**EHCP only**	6.52 (6.37, 6.66)	6.14 (6.03, 6.26)	5.68 (5.55, 5.82)	6.47 (6.37, 6.58)	6.24 (6.18, 6.30)
**CLA only**	6.03 (5.52, 6.55)	8.59 (8.08, 9.10)	8.26 (7.65, 8.88)	9.32 (8.82, 9.81)	8.27 (8.00, 8.53)
**SEN support & CLA**	6.61 (6.32, 6.90)	8.36 (8.09, 8.62)	9.35 (9.01, 9.69)	11.43 (11.14, 11.73)	9.21 (9.06, 9.36)
**EHCP & CLA**	13.03 (12.51, 13.56)	13.93 (13.49, 14.37)	14.25 (13.70, 14.80)	14.93 (14.50, 15.36)	14.14 (13.90, 14.38)
**Mental health related rates**					
**Total**	0.80 (0.79, 0.81)	0.95 (0.94, 0.96)	1.24 (1.23, 1.25)	1.88 (1.87, 1.89)	1.28 (1.28, 1.29)
**No support**	0.65 (0.64, 0.67)	0.68 (0.67, 0.69)	0.88 (0.86, 0.89)	1.35 (1.34, 1.36)	0.94 (0.93, 0.94)
**SEN support only**	0.86 (0.84, 0.88)	1.06 (1.05, 1.08)	1.46 (1.44, 1.48)	2.29 (2.27, 2.31)	1.51 (1.50, 1.52)
**EHCP only**	1.37 (1.30, 1.43)	1.63 (1.57, 1.68)	1.96 (1.88, 2.03)	2.71 (2.64, 2.78)	2.00 (1.96, 2.03)
**CLA only**	1.97 (1.68, 2.27)	5.03 (4.65, 5.41)	5.24 (4.76, 5.72)	6.00 (5.61, 6.40)	4.79 (4.59, 5.00)
**SEN support & CLA**	2.29 (2.12, 2.46)	4.80 (4.60, 5.00)	5.97 (5.70, 6.24)	7.78 (7.54, 8.03)	5.51 (5.39, 5.63)
**EHCP & CLA**	2.90 (2.66, 3.15)	4.40 (4.15, 4.65)	5.58 (5.24, 5.93)	7.27 (6.97, 7.57)	5.28 (5.14, 5.43)

CLA: child looked after; EHCP: education, health and care plan; SEN: Special Educational Needs. Data are presented in accordance with statistical control policy for linked CLA data such that national figures are rounded to the nearest 10.

Appendix Figure 4 - Rate ratios for planned (A), unplanned (B), unplanned mental-health related (C) and pregnancy-related (D) admissions by age and exposure group. Pupils with no support are the baseline group for all rate ratios.

A) Planned Admissions

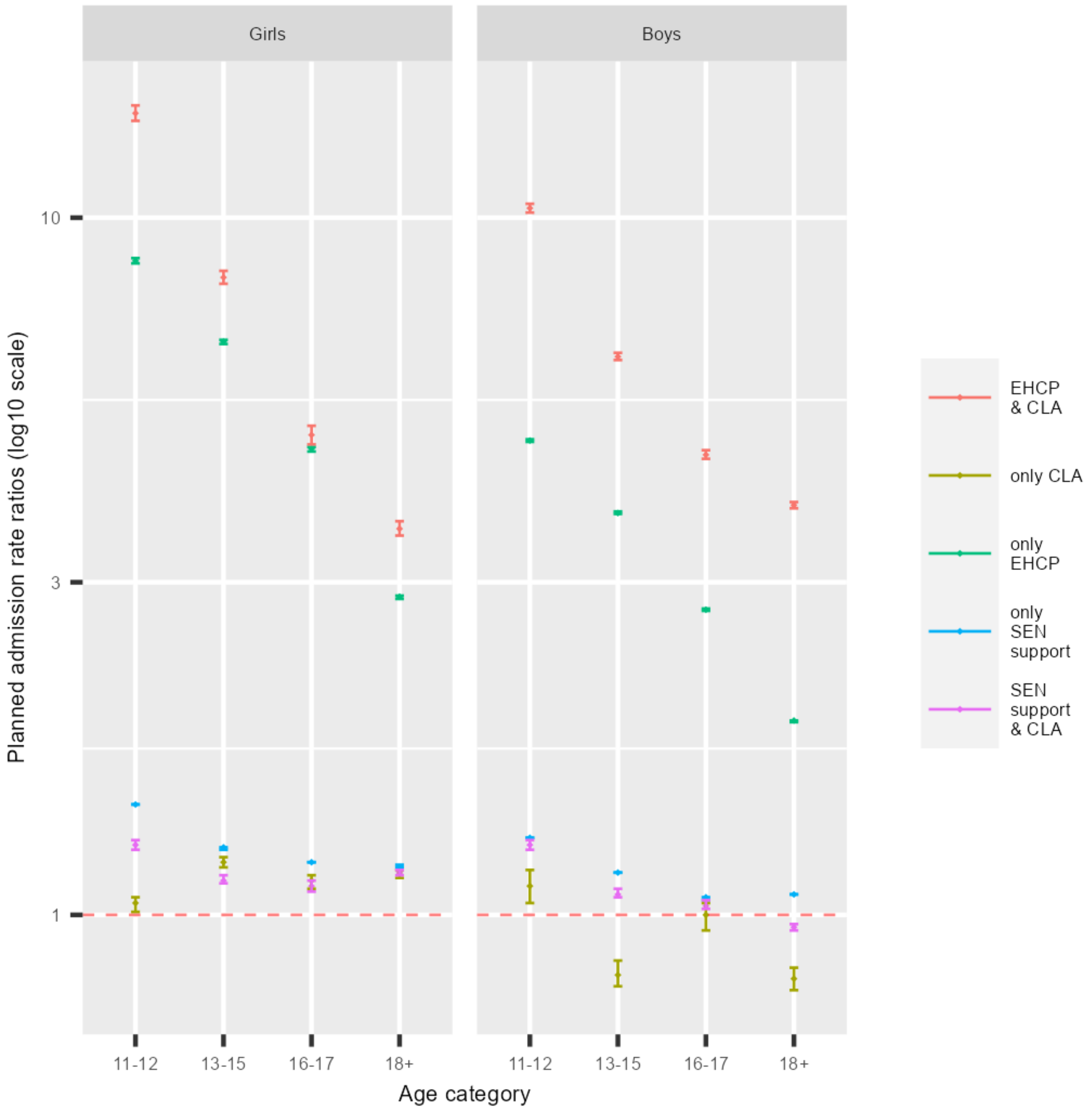

B) Unplanned Admissions

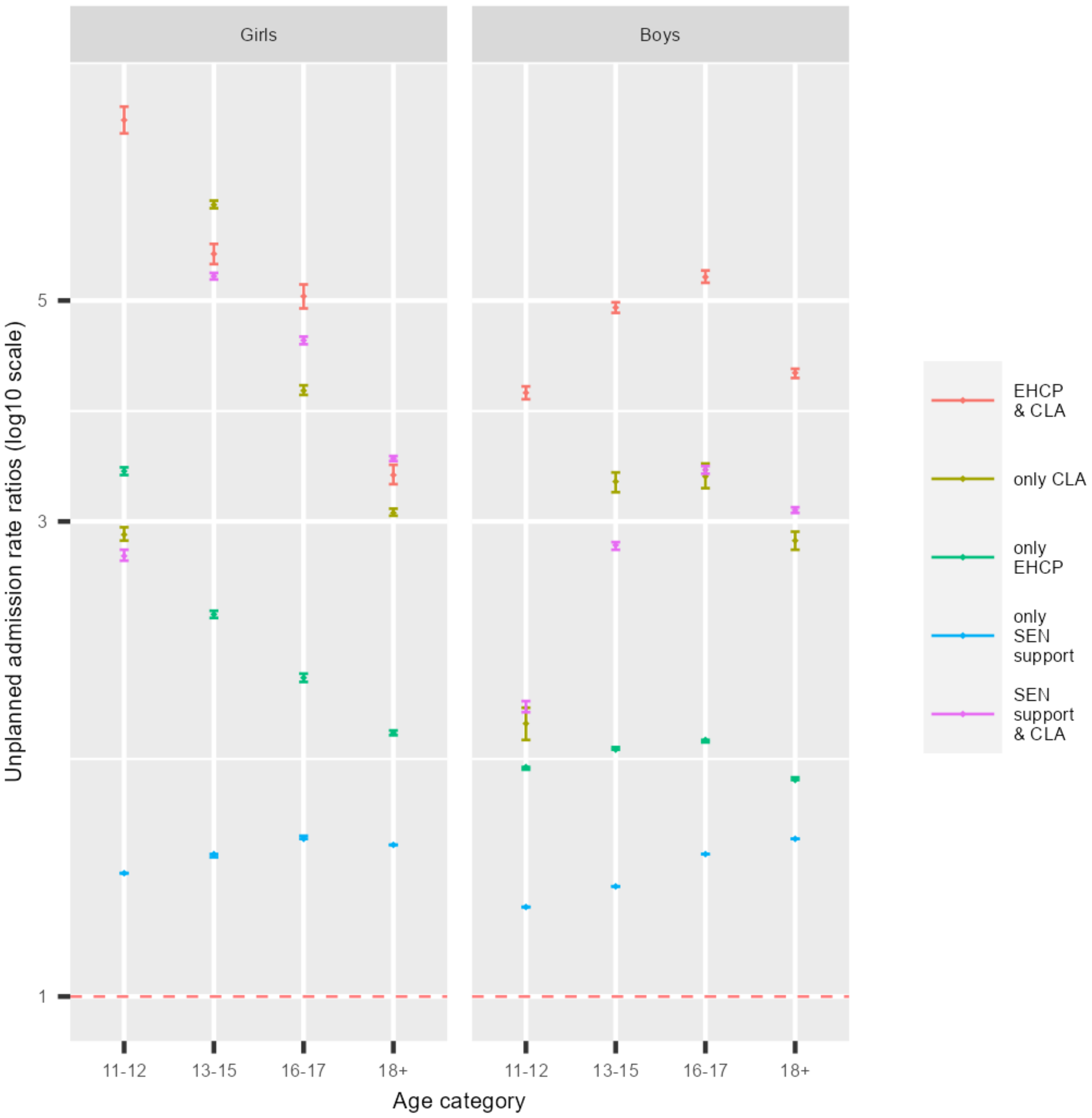

C) Mental health-related admissions

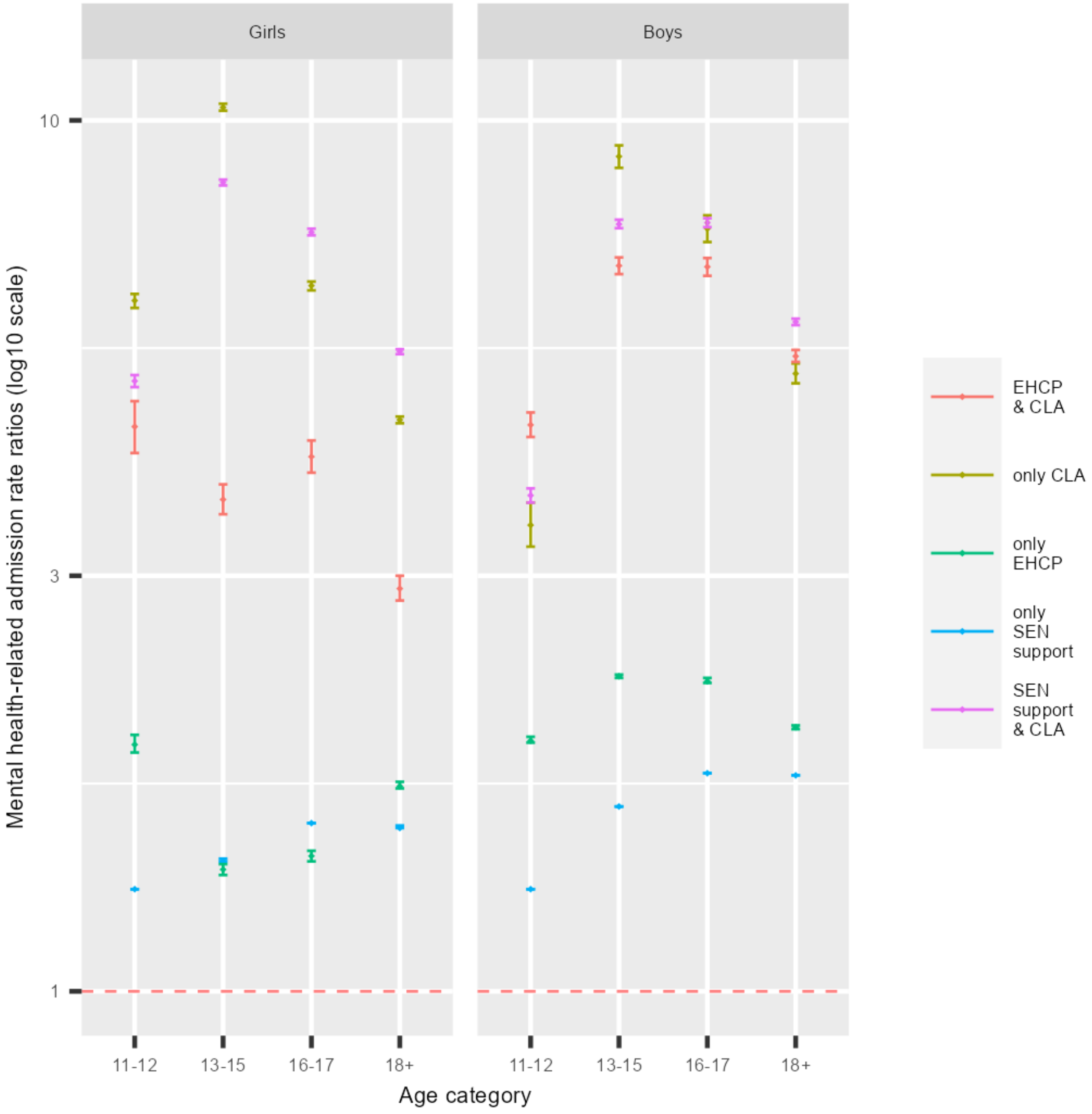

D) Pregnancy-related admissions (girls only)

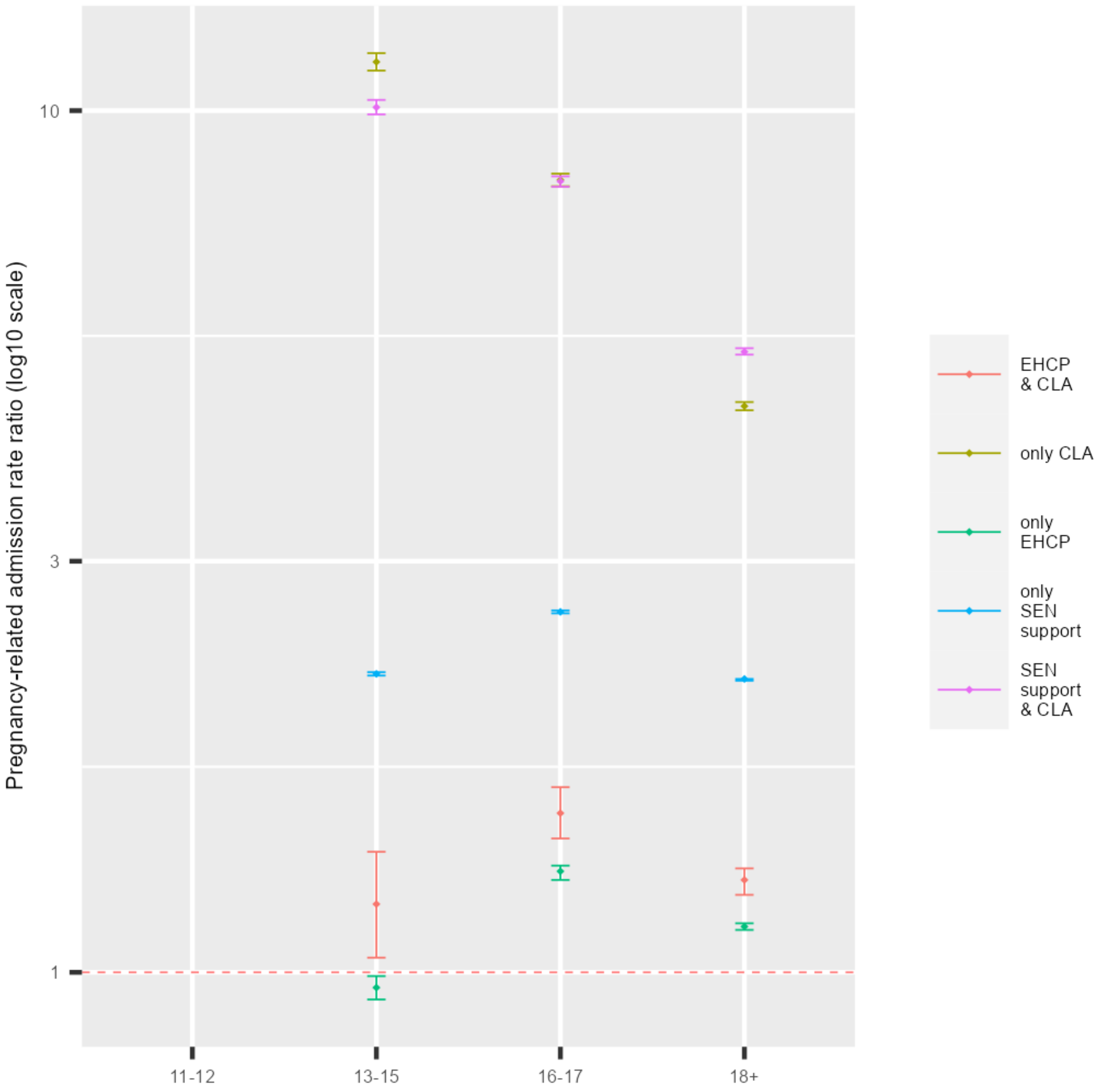

CLA: children looked after, EHCP: education, health and care plan; SEN: Special Educational Needs.
Figure D) Rate ratios for pregnancy-related admissions for girls aged 11-12 years were not estimated due to the small number of events in this age group
